# Generational Differences in Audiometric and Self-Reported Hearing and Hearing Aid Use

**DOI:** 10.1007/s10162-025-00993-2

**Published:** 2025-05-19

**Authors:** Lauren K. Dillard, Lois J. Matthews, Judy R. Dubno

**Affiliations:** Department of Otolaryngology – Head and Neck Surgery, Medical University of South Carolina, Charleston, SC USA

**Keywords:** Hearing loss, Aging, Cross-sectional studies, Cohort studies, Epidemiology, Birth cohort

## Abstract

**Purpose:**

Birth cohort differences capture secular trends in population health. We aimed to determine birth cohort differences, defined by generation, in hearing-related outcomes.

**Methods:**

Participants were from a community-based cohort study. Generation was classified according to birth year: Greatest (1901–1924), Silent (1925–1945), Baby Boom (1946–1964), Generation X (1965–1980), or Millennial (1981–1996) and Gen Z (1997–2012). Primary outcomes were audiometric hearing loss, defined as a worse ear pure-tone average (PTA) of thresholds at frequencies 0.5, 1.0, 2.0, and 4.0 kHz > 25 dB HL, and self-reported hearing difficulty, defined as a score ≥ 6 on the Revised Hearing Handicap Inventory (RHHI). Analyses focused on hearing aid use included only participants with audiometric hearing loss. We used multivariable adjusted logistic regression models to evaluate associations between generation and each outcome. Models were stratified to sex when there was evidence of effect modification.

**Results:**

This cross-sectional study included 1554 participants (mean age 63.7 [SD 14.4] years; 56.8% female, 20.0% racial Minority). The prevalence of audiometric hearing loss, self-reported hearing difficulty, and hearing aid use (among participants with audiometric hearing loss) was 48.9%, 48.8%, and 22.0%, respectively. Generation was associated with audiometric hearing loss in the entire sample and males only. Generation was not consistently associated with self-reported hearing difficulty or hearing aid use.

**Conclusion:**

More recent generations had lower prevalence of audiometric hearing loss. There were no generational differences in self-reported hearing difficulty or hearing aid use. Secular hearing-related trends can inform accurate projections of the burden of hearing loss and health care utilization.

**Supplementary Information:**

The online version contains supplementary material available at 10.1007/s10162-025-00993-2.

## Introduction

As the population ages and life expectancy increases, the caseload of age-related hearing loss is projected to rise dramatically [1]. Hearing loss has serious impacts on individuals, including those related to general health and quality of life [2, 3], and its rising caseload could strain health care systems to provide hearing-related and other relevant services. While the impacts of aging on hearing are well-documented [4–7], there is less evidence on generational or birth cohort differences on hearing [8, 9]. While generation and age are related, generational differences capture positive and negative systematic secular (temporal) differences in exposure to risk factors, which can reflect economic, technologic, and/or social trends that impact population health that are not captured by age alone. Understanding generational differences can (i) ensure accurate prevalence and incidence projections, which can inform health care policy and planning and (ii) provide evidence regarding the success of public health initiatives related to prevention.

Some epidemiological studies reported more recent generations or birth cohorts, defined by birth year, show lower prevalence and 5- and 10-year incidence of hearing loss [8, 9]. Few studies classify generation by participants’ birth year [8–10]. Rather, most studies compare prevalence estimates of hearing loss across several time points (across-cohort) for which hearing data are available [11–14]. Although both approaches capture secular trends in hearing, classifying generation by birth year likely captures systematic differences in exposures more accurately, given that generational classifications are defined by common characteristics or experiences of each group [9, 15, 16]. Some of these studies reported sex differences in generational or across-cohort estimates of hearing loss prevalence [8, 13, 14], but not incidence [9]. Sex differences are plausible and could partially be explained by substantial shifts in the demographic makeup of the workforce, including military service, in the last 100 years.

To date, research has focused on secular changes to hearing measured by pure-tone audiometry, but not on other hearing-related outcomes, including self-reported hearing difficulty or speech perception in noise, both of which complement audiometry. Self-report captures functional impacts of hearing loss not evident from audiometric measures alone and predicts hearing aid uptake and use [17]. Speech perception in noise aims to objectively determine real-world communication ability. Demographic factors, including age, modify relationships between audiometric and self-reported hearing [18, 19], but it is not known whether those relationships vary by generation.

Similarly, few studies have evaluated generational or across-cohort differences in hearing aid uptake or use among people with hearing loss [14, 21]. More recent generations may be more open to adopting hearing assistive technology if they need it, given their general comfort with technology and use of wearable ear-level devices (e.g., earbuds for communication, entertainment). Moreover, recent generations may have experienced improved access to hearing aids, and/or may be more likely to benefit from hearing aids given improvements to hearing aid technology.

Therefore, the purpose of this cross-sectional study, conducted in a community-based cohort of the general population, was to evaluate generational differences in the prevalence of (i) hearing loss, defined by audiometry, self-reported hearing difficulties, speech perception in noise; and (ii) hearing aid use.

## Methods

### Study Population

The Medical University of South Carolina (MUSC) Longitudinal Cohort Study of Age-Related Hearing Loss is an ongoing (1988–current) community-based cohort study based in Charleston, SC, USA [21–27]. Participants must be 18 years or older and in good general health, with no evidence of conductive hearing loss or active otologic or neurologic disease. Participants are continuously enrolled into the cohort.

This cross-sectional study uses data from participants’ baseline examination, which consists of three to six visits that include comprehensive measures of hearing, and health and hearing-related history. The test battery includes pure-tone audiometry, speech recognition measures, and surveys of demographics and general and hearing-related health. Further details on the design of this cohort, and key findings from research conducted in this cohort, are in previous publications [19, 21–27].

At the time of analysis, 1778 participants had baseline data. To be included in this study, participants were required to have complete baseline data for both primary outcomes: audiometric hearing and Revised Hearing Handicap Inventory (RHHI) self-reported hearing difficulty. All protocols for this study were approved by the MUSC Institutional Review Board (approval ID: E-607R) and data were collected under informed written consent.

### Determinant of Interest: Generation

Generation was classified according to birth year, as follows: Greatest Generation (born 1901–1924), Silent Generation (born 1925–1945), Baby Boom Generation (born 1946–1964), Generation X (born 1965–1980), or Millennial (born 1981–1996) and Gen Z (born 1997–2012). Millennial and Gen Z were collapsed into a single group to retain sample size needed for analyses. These classifications reflect commonly used demographic descriptors of generation [9, 15, 16].

### Primary Outcomes: Audiometric Hearing Loss and RHHI Self-Reported Hearing Difficulty

Pure-tone audiometric thresholds at frequencies 0.25, 0.5, 1.0, 2.0, 3.0, 4.0, 6.0, and 8.0 kHz were measured with a clinical audiometer equipped with TDH-39 headphones (Telephonics Corporation, Farmingdale, NY, USA) in a sound-treated booth. All audiological equipment is calibrated to appropriate ANSI standards annually [28]. Thresholds were measured in 5-dB steps following American Speech-Language-Hearing Association (ASHA) standards [29]. All testing was conducted by ASHA-certified audiologists or Au.D. externs under the supervision of the certified audiologists. A pure-tone average (PTA) was calculated from thresholds at 0.5, 1.0, 2.0, and 4.0 kHz in each ear. Hearing sensitivity (as a continuous variable) was defined by PTA in the worse ear, and audiometric hearing loss was defined as a PTA > 25 dB HL in the worse ear [4, 5, 25, 27, 30]. Few participants in this cohort had asymmetrical or unilateral hearing loss [19, 25].

The Hearing Handicap Inventory for the Elderly/Adults (HHIE/A) was administered by paper and pencil prior to audiometric testing, reporting of hearing health history, and participants’ knowledge of hearing-related test results [30–32]. The RHHI was adapted from the 22 items common to the HHIE/A via psychometric analyses (no new questions were added), resulting in an 18-item unidimensional scale of self-reported hearing difficulty [30]. RHHI scores were derived from HHIE/A responses. Like the HHIE/A, response options for the RHHI are yes, sometimes, or no, which are assigned scores of 4, 2, and 0, respectively, and the total score is the sum of all responses. Therefore, RHHI scores range from 0 to 72 points. RHHI self-reported hearing difficulty is defined as a score ≥ 6 points [25].

### Secondary and Supplemental Outcomes: Hearing Aid Use and Speech Perception in Noise

Hearing aid use was treated as a secondary outcome. To be included in analyses focused on associations of generation with hearing aid use, participants were required to have audiometric hearing loss, defined as worse ear PTA > 25 dB HL, and complete data on self-reported hearing aid use. Hearing aid use was defined as self-reported successful hearing aid use, and/or wearing hearing aids at least 2 days per week within 1 year of participants’ baseline examination [22]. Participants classified as Gen X or Millennial/Gen Z were excluded from analyses focused on hearing aid use given limited sample sizes of participants.

Speech perception in noise was treated as a supplementary outcome. To be included in these supplementary analyses, participants must have had Speech Perception in Noise (SPIN) scores within 1 year of their baseline audiogram. Briefly, this outcome was defined by low-context sentence scores on the SPIN test in the worse ear [33, 34], controlled for audibility by the articulation index (AI) speech-audibility metric [21, 34]. Additional methods are in Supplementary Text S1. SPIN scores were treated as a continuous outcome measure.

### Covariates: Demographic Factors

Participants self-reported age, sex assigned at birth (male/female), and race according to US Census Bureau classifications [35]. For this study, race was classified as White or racial Minority to ensure appropriate sample sizes for reporting and analyses. Age was treated as a continuous variable in regression models (described below) and classified as 18 to < 50, 50 to < 60, 60 to < 70, 70 to < 80, and 80 + years for some descriptive analyses.

### Statistical Methods

All analyses were conducted in SAS version 9.4 software (SAS Institute, Inc., Cary, NC).

Demographic differences in sample characteristics between participants who were included and excluded from this study were determined by chi-square for categorical variables and the Kruskal–Wallis test for continuous variables. Statistical significance was defined as *p* < 0.05. For descriptive purposes, we plot relationships of age with PTA and RHHI scores for each generation, overall and stratified by sex, and report the corresponding linear regression coefficients with corresponding 95% confidence intervals (CI).

We used logistic regression models to evaluate associations of generation with the primary outcomes: (i) audiometric hearing loss (PTA > 25 dB HL, worse ear) and (ii) RHHI self-reported hearing difficulty (score ≥ 6 points), and the secondary outcome: (iii) hearing aid use [8, 36]. All (non-stratified) models were adjusted for age, as a continuous variable, sex (female, male), and race (White, racial Minority). Models for RHHI self-reported hearing difficulty and hearing aid use are additionally adjusted for worse ear PTA to evaluate associations of generation with self-reported hearing difficulty, independent of PTA [25]. We determined whether there were sex-specific differences in associations of generation with the primary outcomes by interaction terms and stratified models. We present adjusted sex-stratified logistic regression models when there was evidence of effect modification. Results from all logistic regression models are presented as odds ratios (OR) with corresponding 95% CI. Results from supplementary linear regression models, evaluating associations between generation and SPIN, are presented as regression coefficients with corresponding 95% CI in the supplementary materials.

## Results

Compared to the 1554 participants included in this study, participants who were excluded from this study (*n* = 224), based on lack of outcome data, were more likely to be younger (*p* < 0.01) and have a lower (better) PTA (*p* < 0.01), but did not differ by sex (*p* = 0.81) or race (*p* = 0.18).

Table 1 shows the characteristics of the 1554 participants included in this study. Participants’ mean age was 63.7 (SD 14.4) years, 56.8% were female, and 20.0% were racial Minority (18.9% were Black). The prevalence of audiometric hearing loss and RHHI self-reported hearing difficulty were 48.9% and 48.8%, respectively. The mean PTA and RHHI scores were 27.3 (SD 16.2) dB HL and 10.6 (SD 13.6) points, respectively. Demographic characteristics, including mean age, sex, and race, for each generation, separately, are shown in Supplementary Table S1.

**Table 1:** Study sample characteristics (*n* = 1554)

Characteristics	*n* (%) or mean (SD)
Age (years)	63.7 (14.4)
18 to < 50	213 (13.7%)
50 to < 60	202 (13.0%)
60 to < 70	595 (38.3%)
70 to < 80	430 (27.7%)
80 +	114 (7.3%)
Generation (birth year)	
Greatest (1901–1924)	276 (17.8%)
Silent (1925–1945)	719 (46.3%)
Baby Boom (1946–1964)	377 (24.3%)
Gen X (1965–1980)	92 (5.9%)
Millennial (1981–1996) or Gen Z (1997–2012)	90 (5.8%)
Female sex	883 (56.8%)
Racial minority	310 (20.0%)
Year of baseline examination	
1988–2000	497 (31.9%)
2001–2010	483 (31.0%)
2011–present	578 (37.1%)
PTA, worse ear	27.3 (16.2)
Audiometric hearing loss (PTA > 25 dB HL)	760 (48.9%)
RHHI score	10.6 (13.6)
RHHI self-reported hearing difficulty (score ≥ 6)	758 (48.8%)

Pure-tone average (PTA) averaged thresholds at 0.5, 1.0, 2.0, and 4.0 kHz in the worse ear; *dB HL*, decibel hearing level; *RHHI*, Revised Hearing Handicap Inventory

### Generational Differences in Audiometric Hearing

**Fig. 1:**
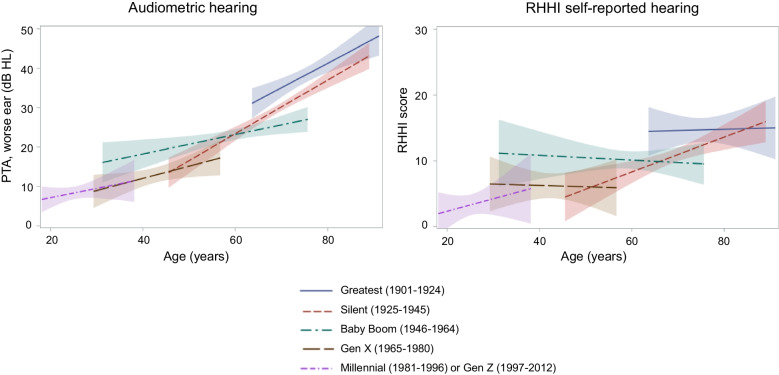
Relationships of age with pure-tone average (PTA) and Revised Hearing Handicap Inventory (RHHI) self-reported hearing difficulty by generation. *dB HL*, decibel hearing level

The prevalence of audiometric hearing loss and mean PTA for each generation and by age group are shown in Table 2. The prevalence of audiometric hearing loss and mean PTA were higher in older generations. In general, both increased with older age. Relationships of age and PTA by generation are in Fig. 1 (left panel). In general, PTA increases as age increases for all generations. Supplementary Figure S1 shows relationships of PTA and age for each generation in males and females, separately. The associations of PTA and age for each generation, overall and by sex, presented as regression coefficients, are in Supplementary Table S2.

**Table 2:** Prevalence of audiometric hearing loss and mean pure-tone average by generation and age group

	18 to < 50 years	50 to < 60 years	60 to < 70 years	70 to < 80 years	80 + years	All ages
Greatest (1901–1924)						
* n*	–	–	71	130	75	276
Prevalence	–	–	67.6%	73.9%	94.7%	77.9%
Mean (SD)	–	–	34.0 (16.8)	37.5 (18.0)	43.8 (11.7)	38.3 (16.5)
Silent (1925–1945)						
* n*	–	35	367	275	39	719
Prevalence	–	37.1%	44.7%	66.9%	89.7%	55.5%
Mean (SD)	–	26.1 (15.3)	25.7 (13.5)	33.1 (14.2)	42.4 (14.7)	29.6 (14.8)
Baby Boom (1946–1964)						
* n*	42	153	157	25	–	377
Prevalence	39.1%	28.8%	39.5%	56.0%	–	36.1%
Mean (SD)	22.9 (16.3)	19.8 (11.4)	24.9 (13.2)	30.3 (14.1)	–	22.9 (13.3)
Gen X (1965–1980)						
* n*	78	14	–	–	–	92
Prevalence	7.7%	7.1%	–	–	–	7.6%
Mean (SD)	12.5 (9.7)	15.2 (8.1)	–	–	–	12.9 (9.5)
Millennial (1981–1996) or Gen Z (1997–2012)						
* n*	90	–	–	–	–	90
Prevalence	3.3%	–	–	–	–	3.3%
Mean (SD)	8.4 (7.6)	–	–	–	–	8.4 (7.6)

Participants in the Silent Generation aged 18–50 were not shown because sample size was < 10. Pure-tone average is defined as the average thresholds at 0.5, 1.0, 2.0, and 4.0 kHz in the worse ear

Associations of generation with audiometric hearing loss in the entire sample, and separately in females and males, are shown in Table 3. Compared to the Greatest Generation, the Silent (OR 0.59, 95% CI 0.42, 0.83), Baby Boom (OR 0.59, 95% CI 0.37, 0.92), and Gen X (OR 0.29, 95% CI 0.11, 0.48). Generations showed lower odds of audiometric hearing loss. For the Millennials and Gen Z group (vs Greatest), the OR was non-significantly lower, and the prevalence of audiometric hearing loss was low (Table 2).

**Table 3:** Associations of generation with audiometric hearing loss. Results are presented as odds ratios with corresponding 95% confidence intervals

	Greatest (1901–1924)	Silent (1925–1945)	Baby Boom (1946–1964)	Gen X (1965–1980)	Millennial (1981–1996) or Gen Z (1997–2012)
Entire sample^a^	REF	0.59 (0.42, 0.83)	0.59 (0.37, 0.92)	0.29 (0.11, 0.48)	0.54 (0.12, 2.34)
Males^b^	REF	0.51 (0.30, 0.90)	0.43 (0.21, 0.88)	0.10 (0.02, 0.45)	0.10 (0.01, 1.12)
Females^b^	REF	0.67 (0.43, 1.04)	0.77 (0.43, 1.38)	0.81 (0.22, 3.03)	2.28 (0.33, 15.50)

^a^Adjusted for age, sex, and race

^b^Adjusted for age and race

*REF*, referent group

In males only, the Silent (OR 0.51, 95% CI 0.30, 0.90), Baby Boom (OR 0.43, 95% CI 0.21, 0.88), and Gen X (OR 0.10, 95% CI 0.02, 0.45) generations, compared to the Greatest Generation, showed lower odds of audiometric hearing loss (Table 3). In males only, the OR for Millennials and Gen Z was non-significant, although the prevalence of hearing loss in that group was low. In females only, generation was not associated with audiometric hearing loss (Table 3). Supplementary Tables S3-4 show the prevalence of audiometric and mean PTA for each generation and age group, and separately in females and males. In general, both males and females show similar trends as those described above for the entire cohort. Prevalence estimates and mean PTA are generally higher for males (vs females) across most age groups, except 80 + years, where estimates are similar.

### Generational Differences in RHHI Self-Reported Hearing

The prevalence of RHHI self-reported hearing difficulty and mean RHHI scores for each generation and by age group are shown in Table 4. In general, the prevalence and mean RHHI scores were higher in older generations. The prevalence and mean scores showed inconsistent patterns across age groups. Relationships of age and RHHI by generation are in Fig. 1 (right panel), and in general, show inconsistent trends across generations. Among participants in the Silent generation only, there is a positive, but small, association between RHHI score and age (Supplementary Table S2).

**Table 4:** Prevalence and mean Revised Hearing Handicap Inventory (RHHI) score by generation and age group

	18 to < 50 years	50 to < 60 years	60 to < 70 years	70 to < 80 years	80 + years	All ages
Greatest (1901–1924)						
* n*	–	–	71	130	75	276
Prevalence	–	–	70.4%	56.2%	68.0%	63.0%
Mean (SD)	–	–	16.4 (15.5)	13.3 (15.2)	15.5 (15.8)	14.7 (15.5)
Silent (1925–1945)						
* n*	–	35	367	275	39	719
Prevalence	–	54.3%	45.8%	56.0%	61.5%	51.2%
Mean (SD)	–	12.3 (14.1)	8.5 (11.5)	12.7 (15.1)	14.7 (17.0)	10.7 (13.7)
Baby Boom (1946–1964)						
* n*	42	153	157	25	–	377
Prevalence	50.0%	39.2%	50.3%	40.0%	–	45.1%
Mean (SD)	12.5 (14.4)	8.6 (12.3)	11.1 (13.9)	9.8 (11.8)	–	10.1 (13.2)
Gen X (1965–1980)						
* n*	78	14	–	–	–	92
Prevalence	35.9%	21.4%	–	–	–	33.7%
Mean (SD)	6.4 (9.4)	5.0 (7.5)	–	–	–	6.2 (9.1)
Millennial (1981–1996) or Gen Z (1997–2012)						
* n*	90	–	–	–	–	90
Prevalence	16.7%	–	–	–	–	16.7%
Mean (SD)	3.3 (7.6)	–	–	–	–	3.3 (7.6)

Participants in the Silent Generation aged 18–50 were not shown because sample size was < 10

Associations of generation with RHHI self-reported hearing difficulty are shown in Table 5. In models adjusted for age, sex, and race, the Silent Generation (OR 0.73, 95% CI 0.54, 0.98) and Millennials and Gen Z (OR 0.33, 95% CI 0.12, 0.86) showed lower odds of RHHI self-reported hearing difficulty as compared to the Greatest Generation. After additional adjustment for PTA, only Millennials and Gen Z showed lower odds of self-reported hearing difficulty (OR 0.24, 95% CI 0.08, 0.73), although the prevalence of RHHI self-reported hearing difficulty was low (Table 4). In separate models for males and females, generation was not associated with RHHI self-reported hearing difficulty (results not shown).

**Table 5:** Associations of generation with Revised Hearing Handicap Inventory (RHHI) self-reported hearing difficulty. Results are presented as odds ratios with corresponding 95% confidence intervals

	Greatest (1901–1924)	Silent (1925–1945)	Baby Boom (1946–1964)	Gen X (1965–1980)	Millennial (1981–1996) or Gen Z (1997–2012)
Base model^a^	REF	0.73 (0.54, 0.98)	0.76 (0.50, 1.14)	0.66 (0.32, 1.34)	0.33 (0.12, 0.86)
Base model + PTA^b^	REF	1.02 (0.71, 1.45)	1.10 (0.68, 1.77)	0.96 (0.43, 2.17)	0.24 (0.08, 0.73)

^a^Adjusted for age, sex, and race

^b^Adjusted for age, sex, race, and pure-tone average (PTA)

*REF*, referent group

### Generational Differences in Hearing Aid Use

Among the 772 participants with audiometric hearing loss at baseline, 726 were eligible for inclusion in secondary analyses focused on hearing aid use, based on the inclusion criteria defined earlier. The mean age of these 726 participants was 70.4 (SD 9.0) years, 46.3% were female and 11.4% were racial Minority, and their mean PTA was 40.9 (SD 11.8) dB HL.

The prevalence of hearing aid use among those 726 participants was 22.0%. The prevalence of hearing aid use for each generation is as follows: Greatest: 22.1%, Silent: 22.1%, and Baby Boom: 21.9%. In participants with audiometric hearing loss, generation was not associated with hearing aid use in models adjusted for age, sex, race, and PTA (Greatest: referent group; Silent: OR 1.42 [95% CI 0.88, 2.28], Baby Boom: OR 1.67 [95% CI 0.80, 3.48]).

### Generational Differences in Speech Perception in Noise

Supplementary Tables S5-6 show the characteristics of the 640 participants included in supplementary analyses, and their mean SPIN scores by generation and age group. Compared to the Greatest Generation, more recent generations showed better speech perception in noise, in the entire sample and in males and females, separately (Supplementary Table S7).

## Discussion

Results from this cross-sectional study, conducted in a community-based sample of the general population, suggest more recent generations were less likely to have audiometric hearing loss, and relatedly, they had better hearing, defined by PTA and SPIN. These trends were observed across age strata. Associations between generation and the prevalence of audiometric hearing loss were present for males, but not females. Results suggest a lack of generational differences in the prevalence of self-reported hearing difficulties or hearing aid use.

The finding that more recent generations had better hearing could be explained by improved population health. For example, more recent generations experienced fewer industrial workplaces and better occupational noise regulations, and military service is less common [37].

Research shows more recent generations experience better outcomes in other age-related conditions, including vision, cardiovascular disease, and cognitive function [15, 38–41]. Shared risk factors between hearing and other age-related conditions, such as improved nutrition, increased availability of vaccinations and medications to reduce infection, and reduced rates of smoking among more recent generations, may improve overall healthy aging [41]. Taken together, generational shifts in health and their association with hearing reiterate that age-related hearing loss may be at least partially modifiable [8, 42].

Most, but not all, previous generation/birth cohort or across-cohort studies reported more recent generations/birth cohorts or cohorts show lower prevalence or incidence of hearing loss [8–10, 12–14, 43]. The study that did not report an association between birth cohort and generation was conducted among 75-year-old people born in 1901, 1915, and 1930 in Sweden [10]. In the present study, those birth years correspond to the Greatest Generation (1901–1924) and beginning of the Silent Generation (1925–1945). Therefore, it is possible that there was not enough variability in birth year to detect an association with hearing loss. In this, and other studies, hearing loss prevalence or incidence is defined by PTA, because it provides a single metric to quantify hearing. Our definition of PTA was comprised of thresholds at frequencies most important for speech understanding. However, this definition does not capture the thresholds at frequencies higher than 4.0 kHz, which is often where damage to the auditory system first occurs [44]. We presented associations of generation with audiometry, SPIN, and self-reported hearing to enhance understanding of generational differences across several hearing-related outcomes.

The finding that generation was associated with the prevalence of audiometric hearing loss in males, but not females, is consistent with previous cross-sectional studies evaluating generational/birth cohort or across-cohort impacts on hearing loss [8, 13, 14, 43]. Associations of generation with audiometric hearing loss were present for males and not females, but associations of generation with speech perception in noise were observed in both sexes. These differences are likely because audiometric hearing loss was modeled as a binary variable, which is meaningful for communicating the burden of hearing loss. However, speech perception in noise does not have a standardized cut-point value to define prevalence and so was modeled as a continuous variable, which could increase statistical power. There are several possible explanations for the sex differences reported in this study. Despite more women entering the workforce in recent generations, men remain more likely to have jobs in noisy environments [45, 46]. Therefore, these sex differences could be explained by policy changes to reduce occupational noise exposure in jobs more likely filled by men. Importantly, among females, the OR for the Silent, Baby Boom, and Gen X Generations, compared to the Greatest Generation, suggested a non-significant trend that more recent generations of females are less likely to experience hearing loss.

To the authors’ knowledge, this is the first study to evaluate generational differences in self-reported hearing difficulty. Previous studies in this cohort showed older individuals, who have a higher prevalence of audiometric hearing loss, are less likely to report hearing difficulties [19, 25]. In this study, generation was not associated with self-reported hearing difficulty. Taken together, results suggest age, rather than the secular constructs captured by generation, is a better predictor of self-reported hearing difficulty.

Results from this study suggested no generational differences in hearing aid use, among Greatest, Silent, and Baby Boom generations. We did not report associations for Gen X and Millennial/Gen Z groups given limited sample sizes of hearing aid users in those groups. While it has been hypothesized that more recent generations, including Baby Boom [47], may be more willing to adopt technology (vs older generations), results suggest that negative perceptions of hearing aids likely persist. Contrary to our findings, a few studies have reported more recent (birth) cohorts may be more likely to use hearing aids [14, 20]. A study conducted in the Norwegian HUNT cohort showed the prevalence of hearing aid use increased from 1996–1998 to 2017–2019 [14]. However, that study evaluated prevalence of hearing aid use in the entire sample, rather than only among those with hearing loss [14]. Another across-cohort study, conducted in NHANES, suggested that among adults aged 50 to 69 years, the prevalence of hearing aid use was higher in 2011–2016 than in 1999–2004 [20]. An association between cohort and hearing aid use was reported only among participants with any hearing loss, but not after stratification to hearing loss severity [20]. These estimates were generated from a limited sample size of 26 and 56 hearing aid users across both cohorts, which was smaller for analyses stratified to hearing loss severity [20]. That limitation, along with the limited research focused on generational/birth cohort effects on hearing aid use, highlights the need for future research on this topic. Understanding generational/birth cohort differences in hearing aid use could inform tailored messaging to reduce stigma and policy that aims to promote treatment for hearing loss across the adult lifespan.

Findings from this study and others [8, 9, 12–14, 43] suggest the caseload of hearing loss may be lower than projected [8]. Research in other areas of aging indicates some generational improvements to health could plateau or reverse due to lack of or negative changes to population health [36]. Examples of factors associated with hearing loss include unmet needs in reducing occupational noise exposure in certain sectors [48], recent declines in nutrition (e.g., consuming more processed foods), increased rates of obesity and sedentary lifestyles [49, 50], and high prevalence of recreational sound exposure [51]. Continued longitudinal epidemiological studies of hearing loss are needed to inform caseload projections and to determine generational trends as more recent generations (e.g., Gen X, Millennials) age. Existing or future systems and policy-level changes, such as availability of over-the-counter hearing aids, improved access to hearing health care, and systematic screening for hearing loss could change generational differences in hearing-related outcomes.

This community-based cohort study has several strengths, including its large and diverse sample of the general population and comprehensive measures of hearing. This cohort study is similar to other epidemiological studies of age-related hearing loss in terms of age and audiometric hearing thresholds, which improves generalizability of study findings [4, 21, 52]. However, some limitations exist. First, although this community-based sample is comprised of individuals from the general population, results may not be generalizable to the entire population, as study participants reside in a relatively small geographic area. Moreover, it is possible there are regional differences in exposure to risk factors for hearing loss, and secular trends in these exposures. Second, despite the relatively large sample size, there were few participants in the Gen X and Millennial/Gen Z Generations with audiometric hearing loss. Similarly, although the proportion of study participants who were Black was consistent with the region's Census data, there were few participants who reported Black race across each generation group. Therefore, we were limited in our ability to draw conclusions related to hearing-related outcomes among Millennial/Gen Z Generations, and we could not report whether generational trends in hearing differed among White and Black, or other, races. Third, cross-sectional observational studies, such as this one, cannot determine temporality, including generational differences in the progression or incidence of hearing loss [9].

## Conclusions

This study, conducted in a diverse, community-based sample of the general population, showed more recent generations have lower prevalence of audiometric hearing loss; these associations were present for males, but not females. Generation was not associated with self-reported hearing difficulty or hearing aid use. Secular trends can inform accurate projections of hearing loss caseload and the burden of hearing loss and health care utilization, including that related to hearing aid uptake.

## Supplementary Information

Below is the link to the electronic supplementary material.

Supplementary file1 (DOCX 232 KB)

## Data Availability

Deidentified participant data are available upon reasonable request to the corresponding author under a data use agreement and institutional approvals according to guidelines of the Medical University of South Carolina.

## Code Availability

Code can be made available upon reasonable request to authors.
