# Supplementary material for: Generational Differences in Audiometric and Self-Reported Hearing and Hearing Aid Use
Source: J Assoc Res Otolaryngol. 2025 May 19;26(4):467–76. doi: 10.1007/s10162-025-00993-2 (PMC12411394; doi:10.1007/s10162-025-00993-2)
Supplement: Supplementary file 1 — Supplementary file1 (DOCX 232 KB) [file 10162_2025_993_MOESM1_ESM.docx]

***Supplementary information for:***

**Generational Differences in Audiometric and Self-Reported Hearing and Hearing Aid Use**

Supplementary text, tables, and figures:

- Supplementary Text S1: Supplementary methods on the measurement of speech perception in noise (SPIN).
- Supplementary Table S1: Demographic characteristics by generation, presented as n (%) or mean (SD).
- Supplementary Figure S1: Relationships of PTA and age for each generation in males and females, separately.
- Supplementary Table S2: Linear regression coefficients (95% confidence interval) for relationships of age with pure-tone average and Revised Hearing Handicap Inventory, separately, for each generation.
- Supplementary Table S3: Prevalence of audiometric hearing loss and mean pure-tone average by generation and age group, in males only.
- Supplementary Table S4: Prevalence of audiometric hearing loss and mean pure-tone average by generation and age group, in females only.
- Supplementary Table S5: Study sample characteristics for supplementary analyses focused on the speech perception in noise (SPIN) test (n=640).
- Supplementary Table S6: Mean speech perception in noise (SPIN) observed – predicted scores by generation and age group.
- Supplementary Table S7: Associations of generation with speech perception in noise (SPIN) observed – predicted scores. Results are presented as linear regression coefficients with corresponding 95% confidence intervals.

**Supplementary Text S1: Supplementary methods on the measurement of speech perception in noise (SPIN)**

Participants underwent the Speech Perception in Noise (SPIN) test.^1^ Fifty-item lists of intermingled high- and low-context sentences were presented in each ear 50 dB above the estimated babble threshold at a +8 dB signal to-babble ratio.^2^ Babble thresholds were estimated based on pure-tone thresholds at frequencies 0.5, 1.0, 2.0, and 4.0 kHz.^3^ Participants repeated the last word of each sentence. To control for audibility, SPIN scores are compared to scores predicted by the articulation index (AI) speech-audibility metric.^4-5^ SPIN scores from low-context sentences only (the more challenging condition) are presented as observed minus predicted values in the worse ear. A positive difference indicates that observed scores are better than predicted and a negative difference indicates scores are poorer than predicted. To be included in these analyses, participants must have had SPIN scores within 1 year of their baseline audiogram. SPIN scores were treated as a continuous outcome measure.

References

1. Kalikow DN, Stevens KN, Elliott LL. Development of a test of speech intelligi­bility in noise using sentence materials with controlled word predictability. J Acoust Soc Am. 1977;61(5):1337–51.
2. Bilger RC, Nuetzel JM, Rabinowitz WM, Rzeczkowski C. Standardization of a test of speech perception in noise. J Speech Hear Res. 1984;27(1):32–48.
3. Bilger RC. Manual for the clinical use of the revised SPIN test. 1984. (Appendix 1).
4. Dubno JR, Lee FS, Matthews LJ, Ahlstrom JB, Horwitz AR, Mills JH. Longi­tudinal changes in speech recognition in older persons. J Acoust Soc Am. 2008;123(1):462–75.
5. ANSI. ANSI S3.5-1997 (R, Methods for calculation of the speech intelligibil­ity index. American National Standards Institute. 2020), (2020). American National Standard Methods for Calculation of the Speech Intelligibility Index (American National Standards Institute, New York). 2020.

**Supplementary Table S1: Demographic characteristics by generation, presented as n (%) or mean (SD).**

|  | Greatest  (1901-1924) | Silent  (1925-1945) | Baby Boom (1946-1964) | Gen X  (1965-1980) | Millennial  (1981-1996) or Gen Z (1997-2012) |
| --- | --- | --- | --- | --- | --- |
| Characteristic |  |  |  |  |  |
| Age (years) | 75.2 (6.6) | 69.1 (6.6) | 59.2 (7.6) | 42.7 (6.9) | 25.3 (4.1) |
| Female sex | 147 (53.3%) | 414 (57.6%) | 212 (56.2%) | 50 (54.4%) | 60 (66.7%) |
| Racial minority | 17 (6.2%) | 99 (13.8%) | 128 (34.0%) | 42 (45.7%) | 24 (26.7%) |

**Supplementary Figure S1: Relationships of pure-tone average and age for each generation in males and females, separately**

**
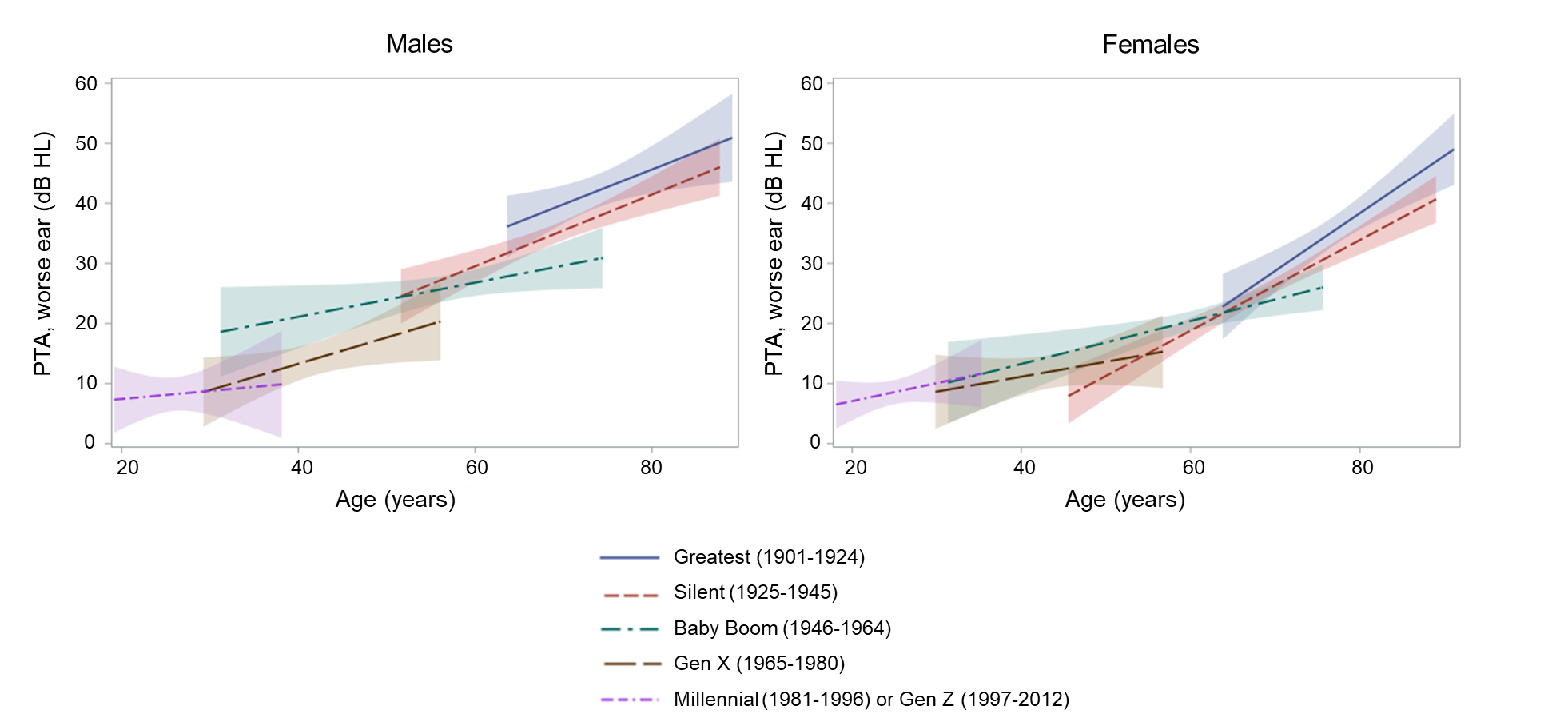
**

Abbreviations: PTA: pure-tone average, dB HL: decibel hearing level

**Supplementary Table S2: Linear regression coefficients (95% confidence interval) for relationships of age with pure-tone average and Revised Hearing Handicap Inventory, separately, for each generation.**

|  | Greatest  (1901-1924) | Silent  (1925-1945) | Baby Boom (1946-1964) | Gen X  (1965-1980) | Millennial  (1981-1996) or Gen Z (1997-2012) |  |
| --- | --- | --- | --- | --- | --- | --- |
| Pure-tone average | | | | | | |
| Entire sample | 0.10  (0.05, 0.15) | 0.13  (0.10, 0.17) | 0.08  (0.02, 0.14) | 0.16  (0.01, 0.31) | 0.07  (-0.04, 0.18) |  |
| Males | 0.09  (0.02, 0.16) | 0.12  (0.07, 0.17) | 0.09  (0.004, 0.18) | 0.24  (0.02, 0.46) | 0.04  (-0.17, 0.25) |  |
| Females | 0.15  (0.09, 0.22) | 0.17  (0.13, 0.22) | 0.12  (0.05, 0.20) | 0.12  (-0.08, 0.32) | 0.08  (-0.05, 0.21) |  |
|  | | | | | |  |
| Revised Hearing Handicap Inventory | | | | | |  |
| Entire sample | 0.004  (-0.05, 0.05) | 0.06  (0.03, 0.10) | -0.01  (-0.07, 0.05) | -0.01  (-0.17, 0.15) | 0.05  (-0.06, 0.17) |  |

**Supplementary Table S3: Prevalence of audiometric hearing loss and mean pure-tone average by generation and age group, in males only.**

|  | 18 to <50 years | 50 to <60 years | 60 to <70 years | 70 to <80 years | 80+  years | All ages |
| --- | --- | --- | --- | --- | --- | --- |
| Greatest (1901-1924) | | | | | | |
| n | -- | -- | 46 | 57 | 26 | 129 |
| Prevalence | -- | -- | 80.4% | 87.7% | 92.3% | 86.1% |
| Mean (SD) | -- | -- | 38.6 (16.9) | 43.2 (17.0) | 45.0 (12.8) | 41.9 (16.3) |
| Silent (1925-1945) | | | | | | |
| n | -- | 19 | 155 | 114 | 17 | 305 |
| Prevalence | -- | 58.0% | 61.3% | 81.6% | 88.2% | 70.2% |
| Mean (SD) | -- | 33.8 (15.7) | 31.2 (13.7) | 38.3 (13.9) | 47.1 (16.3) | 34.9 (14.7) |
| Baby Boom (1946-1964) | | | | | | |
| n | 23 | 78 | 56 | -- | -- | 165 |
| Prevalence | 52.2% | 32.1% | 55.4% | -- | -- | 44.9% |
| Mean (SD) | 28.4 (16.9) | 21.8 (11.2) | 30.0 (14.7) | -- | -- | 26.1 (14.0) |
| Gen X (1965-1980) | | | | | | |
| n | 38 | -- | -- | -- | -- | 38 |
| Prevalence | 5.3% | -- | -- | -- | -- | 5.3% |
| Mean (SD) | 13.0 (8.8) | -- | -- | -- | -- | 13.0 (8.8) |
| Millennial (1981-1996) or Gen Z (1997-2012) | | | | | | |
| n | 30 | -- | -- | -- | -- | 30 |
| Prevalence | 3.3% | -- | -- | -- | -- | 3.3% |
| Mean (SD) | 8.2 (7.4) | -- | -- | -- | -- | 14.0 (9.1) |

Note. Cells with participants < 10 are not shown.

**Supplementary Table S4: Prevalence of audiometric hearing loss and mean pure-tone average by generation and age group, in females only**

|  | 18 to <50 years | 50 to <60 years | 60 to <70 years | 70 to <80 years | 80+  years | All ages |
| --- | --- | --- | --- | --- | --- | --- |
| Greatest (1901-1924) | | | | | | |
| n | -- | -- | 25 | 73 | 49 | 147 |
| Prevalence | -- | -- | 44.0% | 63.0% | 95.9% | 70.8% |
| Mean (SD) | -- | -- | 25.7 (13.3) | 33.0 (17.6) | 43.2 (11.1) | 35.2 (16.2) |
| Silent (1925-1945) | | | | | | |
| n | -- | 16 | 212 | 161 | 22 | 414 |
| Prevalence | -- | 12.5% | 32.3% | 56.5% | 90.9% | 44.7% |
| Mean (SD) | -- | 17.1 (8.7) | 21.7 (11.9) | 29.5 (13.2) | 38.8 (12.5) | 25.7 (13.6) |
| Baby Boom (1946-1964) | | | | | | |
| n | 19 | 75 | 101 | 17 | -- | 212 |
| Prevalence | 21.1% | 25.3% | 30.7% | 47.1% | -- | 29.3% |
| Mean (SD) | 55.8 (15.3) | 17.7 (11.4) | 22.1 (11.5) | 28.5 (14.7) | -- | 20.5 (12.2) |
| Gen X (1965-1980) | | | | | | |
| n | 40 | 10 | -- | -- | -- | 50 |
| Prevalence | 10.0% | 0% | -- | -- | -- | 8.0% |
| Mean (SD) | 12.0 (10.6) | 11.8 (5.9) | -- | -- | -- | 12.0 (9.8) |
| Millennial (1981-1996) or Gen Z (1997-2012) | | | | | | |
| n | 60 | -- | -- | -- | -- | 60 |
| Prevalence | 3.3% | -- | -- | -- | -- | 3.3% |
| Mean (SD) | 8.5 (7.8) | -- | -- | -- | -- | 8.5 (7.8) |

Note. Cells with participants < 10 are not shown.

**Supplementary Table S5: Study sample characteristics for supplementary analyses focused on the speech perception in noise (SPIN) test (n=640).**

| Characteristic | n (%) or mean (SD) |
| --- | --- |
| Age (years) | 62.2 (15.5) |
| 18-<50 | 108 (16.9%) |
| 50-<60 | 96 (15.0%) |
| 60-<70 | 213 (33.3%) |
| 70-<80 | 183 (28.6%) |
| 80+ | 40 (6.3%) |
| Generation (birth year) |  |
| Greatest (1901-1924) | 80 (12.5%) |
| Silent (1925-1945) | 257 (40.2%) |
| Baby Boom (1946-1964) | 204 (31.9%) |
| Gen X (1965-1980) | 43 (6.7%) |
| Millennial (1981-1996) or Gen Z (1997-2012) | 56 (8.8%) |
| Female sex | 359 (56.1%) |
| Racial minority | 131 (20.5%) |
| PTA, worse ear | 26.7 (15.7) |
| Audiometric hearing loss (PTA >25 dB HL) | 309 (48.3%) |
| RHHI score | 10.7 (13.9) |
| RHHI self-reported hearing difficulty (score ≥6) | 310 (48.4%) |
| SPIN observed – predicted, worse ear | -6.4 (17.5) |

Abbreviations: PTA: pure-tone average

**Supplementary Table S6: Mean speech perception in noise (SPIN) observed – predicted scores by generation and age group.**

|  | 18 to <50 years | 50 to <60 years | 60 to <70 years | 70 to <80 years | 80+  years | All ages |
| --- | --- | --- | --- | --- | --- | --- |
| Greatest (1901-1924) | | | | | | |
| n | -- | -- | 22 | 37 | 21 | 80 |
| Mean (SD) | -- | -- | -14.5 (16.2) | -26.7 (20.0) | -31.5 (14.5) | -27.6 (18.7) |
| Silent (1925-1945) | | | | | | |
| n | -- | 11 | 100 | 127 | 19 | 257 |
| Mean (SD) | -- | -11.3 (14.8) | -6.3 (15.8) | -6.1 (15.6) | -19.3 (19.6) | -7.3 (16.2) |
| Baby Boom (1946-1964) | | | | | | |
| n | 16 | 78 | 91 | 19 | -- | 204 |
| Mean (SD) | -13.4 (20.5) | -2.2 (15.1) | -2.6 (14.6) | -2.7 (14.4) | -- | -3.3 (15.5) |
| Gen X (1965-1980) | | | | | | |
| n | 36 | -- | -- | -- | -- | 36 |
| Mean (SD) | 2.4 (12.9) | -- | -- | -- | -- | 2.4 (12.9) |
| Millennial (1981-1996) or Gen Z (1997-2012) | | | | | | |
| n | 56 | -- | -- | -- | -- | 56 |
| Mean (SD) | 5.5 (9.5) | -- | -- | -- | -- | 5.5 (9.5) |

Note. Cells with participants < 10 are not shown.

**Supplementary Table S7: Associations of generation with speech perception in noise (SPIN) observed – predicted scores. Results are presented as linear regression coefficients with corresponding 95% confidence intervals.**

|  | Greatest  (1901-1924) | Silent  (1925-1945) | Baby Boom (1946-1964) | Gen X  (1965-1980) | Millennial  (1981-1996) or Gen Z (1997-2012) |
| --- | --- | --- | --- | --- | --- |
| Audiometric hearing loss | | | | | |
| Entire sample^a^ | REF | 16.98  (12.99, 20.96) | 20.80  (15.94, 25.65) | 25.75  (17.62, 33.88) | 25.69  (15.44, 35.93) |
| Males^b^ | REF | 12.28  (5.94, 18.63) | 16.36  (8.57, 24.15) | 19.50  (6.65, 32.34) | 23.64  (7.84, 39.43) |
| Females^b^ | REF | 20.68  (15.61, 25.75) | 23.75  (17.54, 29.96) | 29.97  (19.44, 40.50) | 26.60  (13.01, 40.19) |

^a^Adjusted for age, sex, and race

^b^Adjusted for age and race
